# Effects of Anthocyanin Supplementation and Ageing Time on the Volatile Organic Compounds and Sensory Attributes of Meat from Goat Kids

**DOI:** 10.3390/ani12020139

**Published:** 2022-01-07

**Authors:** Maria Federica Sgarro, Aristide Maggiolino, Mirian Pateiro, Rubén Domínguez, Francesco Iannaccone, Pasquale De Palo, José M. Lorenzo

**Affiliations:** 1 Department of Veterinary Medicine, University of Bari A. Moro, Valenzano, 70010 Bari, Italy; maria.sgarro@uniba.it (M.F.S.); pasquale.depalo@uniba.it (P.D.P.); 2 Centro Tecnológico de la Carne de Galicia, Rúa Galicia No. 4, Parque Tecnológico de Galicia, San Cibrao das Viñas, 32900 Ourense, Spain; rubendominguez@ceteca.net (R.D.); jmlorenzo@ceteca.net (J.M.L.); 3 Department of Agricultural and Environmental Science, University “Aldo Moro” of Bari, Via G. Amendola, 165/A, 70126 Bary, Italy; francesco.iannaccone@uniba.it; 4 Área de Tecnoloxía dos Alimentos, Facultade de Ciencias, Universidade de Vigo, 32004 Ourense, Spain

**Keywords:** goat meat, red orange and lemon extract, flavonoids, aldehydes, sensory evaluation

## Abstract

**Simple Summary:**

Alternative animal feed sources are being used for the rearing of small ruminants. Among them, the use of agri-food by-products stands out. This strategy meets the demands of consumers towards natural products, offering beneficial effects on meat quality linked to their antioxidant activity properties. In addition, meat ageing allows substantial improvements in palatability attributes, especially tenderness and flavour.

**Abstract:**

The aim of this study was to assess the effects of dietary anthocyanin addition on volatile compounds of meat from goat kids during ageing. For this work, 60 male and female kids were divided into two groups: red orange and lemon extract (RLE group; *n* = 30), which received an RLE extract (90 mg/kg of live weight); and control (CON group; *n* = 30). The phytoextract in dry powder form was rich in bioflavonoids such as flavanones (about 16%) and anthocyanins (about 3%). After slaughtering, the *longissimus thoracis et lumborum* muscle was aged at 4 °C. The volatile organic compound (VOC) and sensorial analyses were carried out at 1, 3 and 7 days. A total of 10 chemical families were identified during the ageing process. Aldehydes were the most abundant VOC, followed by ketones and alcohols. Their contents increased during the process, showing after 7 days of ageing mean values of 20,498, 2193 and 1879 ng/g of meat, respectively. Regarding dietary effects, carboxylic acids, hydrocarbons and thiols presented significant differences between treatments, with higher carboxylic acid contents observed in RLE samples (437 vs. 467 ng/g of meat for CON and RLE batches, respectively; *p* < 0.05). On the contrary, hydrocarbons (436 vs. 254 ng/g of meat for CON and RLE batches, respectively) and thiols (160 vs. 103 ng/g of meat for CON and RLE batches, respectively) displayed significantly (*p* < 0.01) higher amounts in CON compared to the RLE group. Regarding ageing time, the tenderness, juiciness, odour and overall assessment parameters showed significantly higher scores at the end of the whole process (*p* < 0.05). On the other hand, only odour displayed significant differences between treatments, reaching higher scores in CON samples (*p* < 0.05). Therefore, ageing time improved the sensorial properties (tenderness, juiciness, odour and overall assessment) and the VOC content, whereas the inclusion of anthocyanins in the kids’ diet did not have a great impact on the properties of aged meat.

## 1. Introduction

Small ruminant breeding and their meat production and consumption constitute a traditional activity in some marginal and developing countries, although also in the Mediterranean areas [1]. Although meat consumption is mainly linked to ethical, social, and religious factors, the attraction towards this food is influenced by the rheological and sensory properties of meat, as well as by marketing strategies [2]. A great challenge that the meat industry is facing is the increased production costs linked to the negative environmental impacts perceived by consumers [3]. To overcome these problems, many agroindustrial by-products have been proposed as alternative feed sources. In this regard, olive leaves [4], cardon meal, ramie [5], and hydrolysed lignin [6,7,8] have been studied. All these products are well accepted by consumers, since they are perceived as safe and healthy due to their natural origin, their low environmental impact, the possibility of a circular economy application and the presence of bioactive compounds with antioxidant activity that can improve animal welfare and product quality [9].

Many studies have highlighted dietary effects on the chemical, physical and nutritional properties of the meat from small ruminants [10], although also from beef cattle [11] and pigs [12], using different natural antioxidants. Recently, it was also observed that the inclusion of natural extracts with antioxidant activity can modify the volatile compound composition, improving the sensory perception of beef steak [8]. In fact, the flavour of cooked meat arises through a combination of thermally generated aroma volatile compounds (VOCs) and non-VOCs in a matrix of muscle fibres, connective tissues and fat depots [13]. In general, cooked meat is richly flavoured; however, the aroma is strongly influenced by several factors, such as the species, age, chemical composition of the muscle and feeding systems [14,15,16].

Mediterranean Basin countries such as Spain, Italy and Greece are the major European citrus-producing countries. Lemon, orange and mandarin extracts are rich in anthocyanins, which are responsible for the colour of many fruits and also have beneficial antioxidant potential [17]. The red orange and lemon extract (RLE) derived from red orange and lemon processing wastes is rich in flavanones and anthocyanins (further information is available in previous studies) [18,19]. Some studies have reported important improvements in the oxidative status of the muscles in kids [3] and lambs [20] fed with red orange and lemon extract (RLE), with positive effects on their intestinal microflora due to the inhibition of the growth of potentially pathogenic bacteria [21].

Ageing represents an important process, as it leads to the transformation of muscle into meat [14]. The complex reactions that take place during this process can influence the meat quality, especially those attributes related to flavour and odour. Previous studies have shown that ageing improves the eating quality and the release of compounds such as free amino acids or fatty acids, which are the substrates for the formation of flavour compounds [22]. However, most of these studies have been carried out in beef and foal meats [23,24], while little is known about such effects on volatile compounds and sensory quality in lamb. In addition, little is known about the effects of ageing on meat quality of suckling (carcass weight below 7 kg) and light weight lambs (10–13 kg carcass weight), which are commonly consumed in southern Europe [25].

The aim of the present study is to evaluate the effects of dietary supplementation with RLE rich in anthocyanins on the volatile organic compound production and sensory characteristics of meat from goat kids during one week of ageing.

## 2. Materials and Methods

The trial was authorised by the Animal Welfare Body of the University of Naples Federico II (PG/2019/0028161 of 03/19/2019).

### 2.1. Animal Management and Feeding

The experimental procedures were carried out at the experimental farm of the Council for Agricultural Research and Economics, Research Centre of Animal Production and Aquaculture (CREA, Bella Muro, Potenza, Italy). The trial was carried out on 60 male and female Saanen kids. All kids were managed following the procedure described by Salzano et al. [3]. After colostrum intake, all kids of the same age (4 days ± 12 h) and weight (3.1 ± 0.2 g) were divided into two groups: RLE (anthocyanins; *n* = 30), who received an RLE extract (90 mg/kg of live weight) (see below the composition) as an oral food additive; and CON (control; *n* = 30), who received saline. These treatments were given throughout the experimental period, which lasted 40 days. Every day, animals were weighed to record the average weight gain and the RLE extract was mixed with water to obtain a cream [6], which was then administered through a syringe per os. From day 25, animals were maintained in single boxes where they received milk (1 kg/day) three times a day, with 150 g of kids starter (% dry matter (DM): 20.5% crude protein, 1.8% fat, 25% crude fibre) and alfalfa hay *ad libitum* (18.8% crude protein DM, 32.2% crude fibre DM). All kids were weighed (after fasting for 12 h with free access to water). Live weights of 7.87 kg and 7.55 kg were registered for the RLE and CON groups, respectively. The kids were transported to the abattoir located approximately 15 km away, as the travel time was less than 30 min. They were slaughtered on the same day at a European Community-approved abattoir in compliance with European Community laws on Animal Welfare in Transport (1/2005EC) and the European Community regulation on Animal Welfare for the Slaughter of Commercial Animals (1099/2009EC). After slaughtering, meat samples of each kid were randomly assigned to three ageing times (1, 3 and 7 days) for a completely randomised design.

### 2.2. Relative Composition of Red Orange and Lemon Extract (RLE)

The dry powder phytoextract, which was rich in bioflavonoids (flavanones and anthocyanins) and other polyphenols, was obtained according to a patented extraction process (Italian Patent No. 102017000057761) from red orange and lemon processing wastes, exclusively for experimental purpose [3]. The relative concentrations of individual flavanones and anthocyanins were identified by HPLC-PDA-ESI/MS^n^ as described in previous studies [18,19,20]. The individual abundances of each anthocyanin are shown in Table 1.

### 2.3. Meat Sampling

The longissimus thoracis et lumborum muscle was sampled (from the 1st thoracic to the 5th lumbar vertebra) on the slaughter day, after chilling for 4 h. It was cut into three parts and each part was randomly assigned to one of the three experimental storage days: 1, 3 or 7. All sections were placed in extruded polystyrene trays (AERpack PCM0330 produced by Coopbox Italia, Bibbiano, Italy), wrapped in film (Cryovac LID2050, Passirana di Rho, Milano, Italy) and stored until the preassigned storage day at a temperature of 4 °C in the dark as described by Maggiolino et al. [7].

### 2.4. Volatile Compounds (VOC) Analysis

Five-gram meat samples were grilled at 130–150 °C using an electrical griddle (Delonghi, Mod. CG660, Treviso, Italy) until 70 °C was reached in the core. The temperature was measured with a copper–constantan fine-wire thermocouple (Model 5SCTT-T-30-36; Omega Engineering Inc., Norwalk, CT, USA) fixed in the geometrical centres of the samples as described by Maggiolino et al. [13]. After cooking and cooling, samples were minced using a commercial grinder (Moulinex/Swan Holding Ltd., Birmingham, UK).

The volatile compounds were extracted by solid-phase microextraction (SPME) following the procedure described by Maggiolino et al. [8]. The samples were weighed (1 ± 0.05 g) into 20 mL vials (Agilent Technologies, Santa Clara, CA, USA). Before screw-capping the vials with a laminated Teflon–rubber disc, an internal standard (82 ng 2-octanol) was added to the samples to perform a VOC semi-quantitation. The vials were loaded into a Triplus RSH autosampler (Thermo Fisher Scientific, Rodano, Italy), where they were kept at 35 °C for 15 min for equilibration. Once reached, the extractions were carried out at 35 °C for 30 min using a divinylbenzene/carboxen/polydimethylsiloxane 50/30 mm SPME fibre assembly (Supelco, Bellefonte, PA, USA). Then, the fibres were desorbed at 250 °C for 5 min in the injection port of the Trace1300 gas chromatograph (Thermo Fisher Scientific, Rodano, Italy), operating in splitless mode. The gas chromatograph was equipped with an ISQ Series 3.2 SP1 mass spectrometer (Thermo Fisher Scientific, Rodano, Italy). The compounds were separated on a VF-WAX MS capillary column (60 m, 0.25 mm i.d., 0.25 µm film thickness; Agilent, Santa Clara, CA, USA) under the following conditions: injection port temperature, 250 °C; oven temperatures: 35 °C for 5 min, then 1.5 °C/min to 45 °C, then 4 °C/min to 160 °C, and a final increase up to 210 °C at 20 °C/min; the final temperature was held for 7 min. The mass detector was set at the following conditions: detector voltage, 1700 V; source temperature, 250 °C; ionisation energy, 70 eV; scan range, 40–300 amu. Tentative identification of the peaks was achieved using Xcalibur V2.0 software (Thermo Fisher Scientific, Rodano, Italy), in particular Qual Browse, by matching their spectra with the reference mass spectra from the NIST library. The semi-quantitation of the compounds was carried out using the internal standard method, and the amounts are expressed in ng/g.

### 2.5. Sensory Analysis

A panel of ten trained evaluators carried out the sensory analysis test. The selection of the expert assessors was carried out following the British Standards Institution methods [26]. The meat samples for sensory analysis were cut into slices (about 2 cm thick) and cooked as described above for VOC determination. Once the fat and connective tissue were removed, the samples were cut into pieces measuring approximately 1.5 cm^3^, wrapped in pre-labelled foils and placed in a heated incubator until being offered to the evaluators. In order to balance the carryover effects among the meat samples, the tasting test was designed as reported by MacFie et al. [27].

Each expert evaluator conducted ten different sessions, in each of which twelve samples were tasted for a total of 120 meat samples per panellist (two samples for each of the 60 kids). The randomisation of the order of presentation of the samples was carried out through the use of specific sensory analysis software. Tested samples were scored on a 1–10-point scale for tenderness (1 = extremely tough, 10 = extremely tender), juiciness (1 = extremely dry, 10 = extremely juicy), overall assessment (1 = extremely dislike, 10 = extremely like), sweetness, unpleasant taste, meaty odour and unpleasant odours (1 = extremely weak, 10 = extremely strong).

### 2.6. Statistical Analysis

The data set was tested for normal distribution and variance homogeneity (Shapiro–Wilk test). Each kid represented an experimental unit. All data were analysed using the General Linear Model (GLM) procedure in SAS (version 9.3, SAS Institute Inc., Cary, NC, USA), according to the following model:

y_ijk_ = μ + α_i_ + A_j_ + T_k_ + (A × T)_jk_ + ε_ijkl_, (1)

where y_ijk_ are dependent variables, μ is the overall mean, α_i_ is the constant of the random kid effect (i = 1,…60), A is the effect of the jth inclusion of the anthocyanin in the diet (j = 1, 2), T is the effect of the kth ageing (k = 1, …, 3), A × T is the effect of the interaction of the jth anthocyanin inclusion in the diet and kth ageing and ε_ijkl_ is the error term. When not significant, the binary interaction was dropped from the model. A Tukey test for repeated measures was applied to evaluate the differences according to ageing time, setting the significance at *p* < 0.05.

## 3. Results and Discussion

### 3.1. Volatile Organic Compounds

Table 2 shows the effects of anthocyanin diet addition on total volatile organic compounds of meat from goat kids. A total of 53 volatile substances were isolated and identified using SPME/GC-MS. The obtained compounds were divided into ten families according to their chemical nature: 12 aldehydes, 11 alcohols, 11 ketones, 8 carboxylic acids, 5 hydrocarbons, 2 furans, 1 aromatic hydrocarbon, 1 lactone, 1 sulphur compound and 1 thiol. However, there are some chemical families, usually identified in beef and horse meat, that were not found in the present study, which could be due to the different types of cooking carried out on the samples [23]. This is the case for esters [28] and nitrogen compounds [29]. Aldehydes were the main compounds identified, followed by ketones and alcohols. Aldehydes were also the main VOCs originated from cooked donkey, foal, lamb kids and pork meat [14,15,16,30,31].

Dietary anthocyanin addition did not have a significant effect on alcohols, aldehydes, furans, lactones, sulphur compounds or ketones (*p* > 0.05). Similar results were observed in the meat of lambs fed with olive cake and linseed [30]. Regarding hydrocarbons and thiols, CON groups showed significantly (*p* < 0.01) higher values than those observed in RLE samples (436.2 vs. 254.7 ng/g of meat and 160.1 vs. 102.6 ng/g of meat for hydrocarbons and thiols after 7 ageing days, respectively). The opposite behaviour was observed for carboxylic acids whose contents were higher in RLE (467.0 vs. 436.9 ng/g of meat for RLE and CON groups, respectively).

Except for sulphur compounds, VOCs were affected by ageing time. In this regard, the values increased with ageing time. This trend was also observed by other authors, who confirmed that refrigerated ageing for 7–30 days increased the flavour of beef [32,33]. Regarding aldehydes, aromatic hydrocarbons, carboxylic acids and lactones, this increase was only significant in the CON group (*p* < 0.05), while in furans this effect was observed only in the RLE group (*p* < 0.05).

#### 3.1.1. Aldehydes

Aldehyde variations due to oral anthocyanin administration and ageing time are shown in Table 3. This chemical family is one of the main ones identified in ruminant meat [34]. The CON group showed an increasing trend for pentanal, hexanal, heptanal, nonanal, 2-nonenal, octanal, 2-octenal, 2,4-decadienal and 2,4-dodecadenial (*p* < 0.05) contents. In contrast, no variations in these compounds were observed in the RLE group (*p* > 0.05). Hexanal was the most abundant aldehyde of the two groups, similarly to what was reported for meat samples from lamb kids by other authors [31], followed by nonanal, heptanal and octanal, although their contents were much lower. Hexanal, considered as the greatest indicator of lipid oxidation in meat [35], increased throughout the ageing process. A similar behaviour was observed in other ageing studies carried out with beef and foal meat [13,33]. However, the changes observed during maturation were more significant than those found by other authors. These differences could be related to the fact that in most cases ageing is carried out under vacuum conditions [23]. Therefore, the changes that occur under these conditions are usually minimal due to the slight lipid autoxidation.

Regarding the origin of this compound, hexanal derives from oleic, linoleic and arachidonic acids [36]. Therefore, the feeding of the animals would influence its content. However, the higher values were obtained in the CON group, whose values increased significantly by 53% until the end of the process, while the RLE group increased by 29%, although no significant differences were observed between groups. This could be due to the protective effect that RLE exerts against lipid and protein oxidation [3]. This is especially important at the sensory level, since this compound is usually associated with highly aromatic notes (freshly cut grass and green aromas at low levels, or unpleasant rancid aromas at high levels), even at low concentrations, due to its low odour detection threshold [36]. Therefore, the RLE addition in the feeding of the animals would minimise the appearance of off-flavours, favouring the sensorial acceptability of the meat derived from these animals. Similar results were found by Maggiolino et al. [8] when the Limousine steers’ diet was supplemented with *Pinus taeda* hydrolysed lignin. Contrary to these results, Vasta et al. [37] observed that the inclusion of *Rosmarinus officinalis* or *Artemisia herba alba* essential oils into the lambs’ diet did not have a significant effect on the volatile compounds profile. No effect was also found by Del Bianco et al. [38] when tannin extracts obtained from mimosa (*Acacia mearnsii*), chestnut (*Castanea sativa*) or tara (*Caesalpinia spinosa*) were added to the diets of Sarda × Comisana lambs.

The same behaviour was observed in other oxidation markers derived from the lipid oxidation of oleic acid, such as heptanal, octanal and nonanal [39], which displayed significantly higher values (*p* < 0.01) in CON meat. In the case of octanal, this effect was observed from the first day, while in the case of nonanal and heptanal we had to wait 3 and 7 days, respectively. Although the partial replacement of conventional lamb feedstuffs by olive cake and linseed did not significantly affect any of the compounds derived from lipid oxidation, higher contents were also obtained in conventional cereal-based concentrates [30]. In the case of heptanal, the diets that contain stoned olive cake or with rolled linseed and stoned olive cake exceeded the contents obtained in the control diet (4.105 and 4.182 vs. 3.883 log_10_ specific ion peak area units, respectively) [30]. Regarding the aromatic notes associated with these compounds, they usually provide pleasant meaty notes to the product [36]. Regarding minority aldehydes, propanal and benzaldehyde increased in both groups during ageing (*p* < 0.01), although the first one showed higher values (*p* < 0.01) in the CON group compared to the RLE group after 7 days of ageing (45.94 vs. 39.03 ng/g of meat, respectively). After 7 days, 2,4-dodecanal (*p* < 0.01) also showed higher values in CON meat. Moreover, CON meat displayed higher values of 2-nonenal compared to RLE at 3 days (*p* < 0.05) and 7 days (*p* < 0.01) of ageing.

#### 3.1.2. Ketones

The effects of anthocyanin diet addition and ageing on ketones of meat from goat kids are reported in Table 4. 

The main compound identified was 6,7-dodecanedione. Although they were much lower, the contents of 2,3-pentanedione and 1-octen-3-one also stood out. In fact, 1-octen-3-one is considered together with hexanal and octanal as one of the main compounds identified in ruminant meat [37]. The total ketones increased with ageing, showing the highest values at 7 ageing days. For 2-hexanone, 2-heptanone, 3-hepten-2-one, 5-hepten-2-one,6-methyl, 3,5-octadien-2-one and the 6,7-dodecanedione, the ageing time resulted in significant increases in both CON (*p* < 0.01) and RLE (*p* < 0.05) groups. For 2,3-pentanedione, 2-heptanone,6-methyl-, 2-octanone and 3-nonanone, such increases were only significant (*p* < 0.01) in the CON group. The same behaviour was observed for 1-octen-3-one (26.64, 34.21 and 58.50 ng/g of meat at 1, 3 and 7 days of ageing, respectively; *p* < 0.05), which was only identified in the CON group. This tendency to increase during ageing was also found by Insausti et al. [40], who observed higher ketone contents during aging. However, this was only significant in two (2-heptanone and 3-octanone) of the four compounds identified. This effect could be due to the fact that the meat was aged under an oxygen-permeable film, which would favour the interactions between protein oxidation products and some amino acids. On the contrary, this behaviour was hardly observed in other species matured under vacuum conditions. In this regard, only 2,3-pentanedione was significantly modified during the ageing of donkey meat [14], while 2-heptanone, 3-heptanone and 2-butanone increased (until day 6) in foal meat [13,15].

Regarding the feeding effect, 6,7-dodecanedione, 5-hepten-2-one,6-methyl-, 2-hexanone, 4-methyl-, 3-hepten-2-one, 2-octanone and 3-nonanone were affected by the inclusion of RLE in the diet. At the end of ageing, 2-octanone, 3-nonanone and 3,5-octadien-2-one showed higher values in CON samples compared to the RLE group (*p* < 0.01). Moreover, the CON group showed significantly higher contents of 3-hepten-2-one and lower values of 2-hexanone (*p* < 0.05). In addition, it is important to highlight the contents of 2-heptanone, which could be used as biomarkers of lipid degradation (linoleic acid (C18:2*n*-6) oxidation). In fact, it is common to find this compound in ruminants fed with commercial concentrates [41,42]. In the present study, although no significant differences were found between groups, slightly higher contents were found in CON samples (26.68 vs. 22.18 ng/g of meat, respectively). Moreover, this compound is related to the flavour of lamb due to its low detection odour threshold and its peculiar aroma (butter and cheese notes, spicy) [43,44].

Despite the significant results mentioned above, hardly any significant interactions were detected between the main fixed factors (diet × ageing time), except for 3-hepten-2-one, 3,5-octadien-2-one and 3-nonanone.

#### 3.1.3. Alcohols

Table 5 shows the effects of the inclusion of RLE in the diet and ageing time on alcohol contents of longissimus thoracis et lumborum muscle. Alcohols production were affected by ageing, and an increasing trend was observed as the ageing process increased. The 1-pentanol, 1-hexanol,2-ethyl-, 1-heptanol and 1-octanol increased during ageing in both CON (*p* < 0.01) and RLE meat (*p* < 0.05) groups. The same behaviour was observed in 1-octen-3-ol (*p* < 0.05), which is consistent with the results found by Insausti et al. [40] in Navarra breed lamb meat aged for 4 days. On the other hand, 1-penten-3-ol, 2-pentene-1-ol and 1-hexanol increased only in the CON group (*p* < 0.05). The same happened with 2-hexen-1-ol (*p* < 0.01), which was identified only in the CON group. Conversely, 1-nonanol did not change during ageing. This alcohol and 2-penten-1-ol showed the lowest contents.

Taking into account the total contents, 1-octen-3-ol was the alcohol identified in the highest quantity, followed by 1-pentanol and 1-hexanol. These are considered products of lipid oxidation. For 1-octen-3-ol and 1-pentanol, the origin is the linoleic acid degradation, while 1-hexanol is a product of the oxidation of oleic acid [35]. These compounds have also been identified as the most abundant in aged lamb, donkey and foal meats [14,15,40]. However, the contents obtained in the present study were higher than those found in other aged meats, probably due to the different maturation method used.

These alcohols together with 1-heptanol and 1-octanol have an important role in the final flavour of the meat, although their contribution to volatile flavour is less than other volatiles due to their high odour threshold [36]. Grassy and mushroom-like odours distinguish 1-octen-3-ol, while sweet or fruity distinguish 1-pentanol and herbal and fatty notes distinguish 1-hexanol [45].

The volatile alcohol profile was not affected by anthocyanin supplementation. This outcome was also observed in the meat obtained from plant-fed lambs despite the antioxidant properties associated with these plants [37]. 1-Nonanol was the only compound that was affected by the inclusion of RLE in the diet of the animals, showing higher values than in CON meat (2.94 vs. 10.73 ng/g of meat; *p* < 0.01). In the same way, no significant interactions were observed between the main fixed factors (diet × ageing time).

#### 3.1.4. Hydrocarbons and Carboxylic Acids

Table 6 shows the effects of ageing and RLE on hydrocarbons and carboxylic acid volatile compounds of meat from goat kids. Regarding hydrocarbons, only 6 compounds were detected in the meat of kid lambs. These compounds were grouped into 2 linear, 2 branched, 1 cyclic and 1 aromatic hydrocarbon. In terms of their contribution to the aroma of meat, these compounds can be divided into two groups: linear, branched and cyclic hydrocarbons, which hardly contribute to the flavour of the meat due to their high odour threshold; and aromatic hydrocarbons, which on the contrary provide important aromatic notes due to their low odour threshold [45].

Aromatic hydrocarbons are less present in meat than linear hydrocarbons [13]. Unlike other studies carried out with the same type of meat and under the same ageing conditions (over-wrapped with a commercial transparent film) [40], only one aromatic hydrocarbon was found. Benzene 1,3-dimethyl was identified only in low concentrations in the CON group and increased after 7 ageing days (*p* < 0.01). However, in most studies it was the main volatile compound identified [15,40,42]. The fact that it has not been identified in the RLE group could be related to the protective effect that the inclusion of RLE in the diet of animals would have against lipid oxidation, preventing the generation of this oxidation product [46].

Regarding carboxylic acids, their contribution to the total volatile compound profile was low, which is in agreement with previous studies conducted in lamb meat [42,47]. This scarce presence in muscle would be related to the fact that branched chain fatty acids tend to be deposited in adipose tissue [48]. In the present study, hexanoic and heptanoic acids were the main carboxylic acids identified, although important contents were also observed for ethanoic acid and nonanoic acid. The contents of carboxylic acids increased with ageing, although only for pentanoic, hexanoic, octanoic and nonanoic acids were the results significant. In this regard, after 7 days there were increases in pentanoic acid (*p* < 0.01) and heptanoic acid (*p* < 0.05) in the CON group. For octanoic acid and nonanoic acid, significant differences (*p* < 0.05) were observed in both groups. A similar trend was observed in aged horse meat, although in these cases less compounds were identified (butanoic acid, hexanoic acid, formic acid, octyl ester) [13,14].

Within this family of volatile compounds, it is important to highlight 4-methyloctanoic, 4-ethyloctanoic and 4-methylnonanoic acids, since they contribute to the characteristic mutton-like aroma [49]. In the present work, these compounds were not identified. In contrast, nonanoic acids were detected, which are related to unfavourable odours, such as fatty and goaty odours [50]. However, the inclusion of RLE in the diet decreased the contents of this compound (45.55 vs. 51.14 ng/g of meat for RLE and CON, respectively), avoiding possible rejection, since its presence in the meat could compromise the acceptability of the consumer. This effect was also observed in pentanoic acid (4.19 vs. 9.97 ng/g of meat for RLE and CON, respectively; *p* < 0.01) and heptanoic acid (54.01 vs. 161.03 ng/g of meat for RLE and CON, respectively; *p* < 0.05), which showed significantly lower values in the RLE group after 7 days of ageing. This acid release was also lower in steers and lambs whose diet was supplemented with hydrolysed lignin or tannins [8,38].

#### 3.1.5. Other Compounds

Furans, lactones, sulphur compounds and thiols detected in meat from goat kids are shown in Table 7. Regarding the furan family, only two compounds were identified, furan, 2-ethyl- and furan, 2-pentyl-. No significant effect of diet or ageing time was observed (*p* > 0.05). However, slightly higher values were detected in the mentioned compounds as maturation progressed. Moreover, the samples from the RLE group presented higher values than those obtained from CON (16.20 vs. 15.22 ng/g of meat for furan, 2-ethyl-, and 20.28 vs. 14.44 ng/g of meat for furan, 2-pentyl-). In the same way, Vasta et al. [37] did not find a significant effect on volatile compound profiles of Barbarine lambs when the diet of the animals was supplemented with essential oils of rosemary and artemisia. In this case, the contents of these volatile compounds were lower in treated samples. Therefore, in the present study, the inclusion of RLE did not have an antioxidant effect on the production of furan, 2-pentyl-, which was a result of the degradation of C18:2*n*-6 [35]. Regarding the aromatic notes provided by these volatiles (green bean and butter flavours) [51,52], it is to be expected that their contribution to the aroma of meat from goat kids will not be very prominent, since they represented a small percentage of the total volatile compound profile.

Similar contents were found for lactones, although only a single compound was identified. Butyrolactone was also detected in other studies conducted in lamb meat. However, in these studies this compound was included in the ketone family [41]. This volatile compound, which has a low perception threshold, is associated with caramel and sweet odours [52]. Regarding the origin of this compound, some authors affirm that it would be related to the oxidation of hydroxy-fatty-acids in the rumen [47,53]. In this regard, the feeding of the animals would influence the contents of this compound. However, in the present study and according to the results found by other authors [41], the diet of the animals did not affect the contents of this lactone (12.03 vs. 10.59 ng/g of meat for CON and RLE, respectively). Contrary to these outcomes, some authors found higher values in lambs fed concentrate-based diets than those finished on pasture or grass [54]. On the other hand, an increase in the contents was observed as the days of ageing increased, although it was only significant (*p* < 0.01) in the CON group.

Regarding sulphur compounds, these compounds contribute to the general aroma of meat and can add undesirable flavours and odours [55]. This is the case for dimethyl sulfone, identified in the present study, which is associated with sulphur and burnt odours [52]. However, no significant differences were found in any of the evaluated factors. This volatile was also identified by other authors in lamb meat [47].

### 3.2. Sensory Evaluation

The results of the sensory evaluation are reported in Table 8. The obtained results showed a greater effect of ageing on the sensory attributes evaluated. Tenderness, juiciness, meaty odour and overall assessment showed significant differences throughout the ageing process. The scores obtained for these attributes increased with ageing time. In this regard, tenderness and juiciness were characterised by higher values after 7 ageing days in both experimental groups (*p* < 0.05). As expected, in the case of tenderness, the higher scores obtained at the end of ageing give an idea of the positive effect that maturation has on the texture parameters of meat. This was corroborated by the results found by Salzano et al. [3], who observed a decrease in shear force values during ageing (26.29, 23.02 and 21.02 N for days 1, 4 and 7 of ageing, respectively) in meat from goat kids. On the contrary, these attributes were not significantly affected by ageing in aged lamb and donkey meat [14,40]. In this regard, Insausti et al. [40] hardly observed an effect of ageing on the flavour or odour attributes of lamb meat from the Navarra breed. Regarding the overall assessment, ageing has a positive effect, since the obtained scores increased during maturation.

On the other hand, the sensory evaluation was hardly affected by diet supplementation. Meaty odour was the only parameter that showed significant (*p* < 0.05) differences between samples; the values were higher in CON group compared to the RLE meat (8.12 vs. 7.45 at 7 days of ageing, respectively). This result was in agreement with the outcomes observed by other authors in the meat from lambs whose diet was supplemented with tannin extracts obtained from mimosa, chestnut, tara or grape seed extract [38,56]. This lack of sensory differences among treatments could be related to the short feeding times and the low intramuscular fat contents in young lambs, being that fat depots are the principal sources that contribute to meat flavour and odour [40,57]. In addition, these results would also reflect the protective effect that RLE extracts have against lipid oxidation and the formation of aldehydes, the main contributors of the meat aroma [35]. Finally, the same effect was observed in the overall assessment, which was not affected by the diet of the animals (8.02 and 8.10 for CON and RLE, respectively).

## 4. Conclusions

The outcomes from the present paper increase the knowledge of the effects of ageing in meat from goat kids. Ageing increased the VOC profile, which resulted in an important effect on the meat aroma. A sensorial analysis showed that ageing might increase the sensory odour and flavour quality in meat, since increases were observed throughout maturation. Moreover, a positive effect on sensory attributes highly valued by consumers (tenderness and juiciness) was also observed. Aldehydes were the main compounds identified in meat from goat kids. The incorporation of RLE in the diet of the animals did not have a great effect on VOCs derived from lipid oxidation, probably due to the short time of administration. Therefore, further trials should be conducted to better understand the potential effect of this product on the VOC and sensory profiles of meat from goat kids.

## Figures and Tables

**Table 1:** Anthocyanin profile of red orange and lemon extract (RLE).

Compound	[M]^+^ (*m*/*z*)	MS^n^ (*m*/*z*)	Anthocyanin	Relative Composition (%) ^(a)^
1	611	449/287	cyanidin 3,5-diglucoside	1.29
2	465	303	delphinidin 3-glucoside	2.67
3	611	287	cyanidin 3-sophoroside	0.41
4	449	287	cyanidin 3-glucoside	39.97
5	595	287	cyanidin 3-rutinoside	1.30
6	479	317	petunidin 3-glucoside	1.59
7	551	465/303	delphinidin 3-(6″-malonyl)glucoside	1.43
8	463	301	peonidin 3-glucoside	2.98
9	565	479/317	petunidin 3-(6″-malonyl)glucoside	1.45
10	535	449/287	cyanidin 3-(6″-malonyl)glucoside	21.76
11	-	271	pelargonidin derivative	1.44
12	549	463/301	peonidin 3-(6″-malonyl)glucoside	13.80
13	-	287	cyanidin derivative	2.39
14	-	301	peonitin derivative	1.82
			Total anthocyanins (g CGE/100 g)	2.66

[M]^+^ (*m*/*z*): Mass peak; MS^n^ (*m*/*z*): MS fragmentation model; ^(a)^ relative composition of anthocyanins calculated from peak areas recorded at 520 nm. The total anthocyanin content was expressed as mg of cyanidin 3-glucoside equivalents (CGE)/100 mL.

**Table 2:** Effects of anthocyanin diet addition and ageing on volatile organic compounds of meat from goat kids (*n* = 30 samples for each experimental group). Results are expressed as ng/g of meat.

		Ageing Time						
	Group	Day 1	Day 3	Day 7	SEM ^1^	*p*-Value		
						A ^2^	T ^3^	A × T
Alcohols	CON	1280.53 ^A^	1489.32 ^A^	1895.18 ^B^	151.95	0.7308	0.0019	0.9063
	RLE	1344.01 ^a^	1587.39 ^a^	1862.70 ^b^				
Aldehydes	CON	14,805.32 ^A^	18,802.15 ^AB^	22,655.74 ^B^	1615.46	0.1180	0.0027	0.4600
	RLE	14,463.84	17,173.54	18,339.32				
Aromatic hydrocarbons	CON	10.79 ^A^	12.57 ^AB^	13.93 ^B^	0.73	-	0.019	-
	RLE	^-^	^-^	^-^				
Furans	CON	22.37	26.10	29.67	3.23	0.0663	0.0260	0.8643
	RLE	25.73 ^a^	30.75	36.49 ^b^				
Carboxylic acids	CON	220.09 ^A^	255.48 ^a, x^	436.88 ^Bb^	49.60	0.0196	0.0030	0.4844
	RLE	336.62	401.20 ^y^	466.98				
Hydrocarbons	CON	201.96 ^A,x^	255.09 ^A,X^	436.22 ^B,X^	14.68	<0.0001	<0.0001	<0.0001
	RLE	159.99 ^Aa,y^	192.39 ^Ab,Y^	254.74 ^B,Y^				
Lactones	CON	5.59 ^A^	7.03 ^A^	12.03 ^B^	1.13	0.419	<0.001	0.252
	RLE	7.44	8.88	10.59				
Sulphur compounds	CON	14.78	18.46	31.58	9.41	0.176	0.308	0.933
	RLE	26.44	31.79	38.19				
Thiols	CON	80.34 ^A^	93.61 ^A^	160.07 ^B,X^	6.48	<0.0001	<0.0001	<0.001
	RLE	71.36 ^A^	85.85 ^AB^	102.55 ^B,Y^				
Ketones	CON	1111.80 ^A^	1303.12 ^A^	2228.34 ^B^	198.01	0.0972	0.0001	0.3172
	RLE	1497.73 ^a^	1806.33	2157.54 ^b^				

^1^ Standard error of the means; ^2^ anthocyanin; ^3^ ageing time. Different letters in the same row show statistical differences during time in the same group: ^A,B^ = *p* < 0.01; ^a,b^ = *p* < 0.05. Different letters in the same column show statistical differences between groups at the same ageing time: ^X,Y^ = *p* < 0.01; ^x,y^ = *p* < 0.05. CON: control; RLE: red orange and lemon extract.

**Table 3:** Effects of anthocyanin diet addition and ageing on aldehyde volatile compounds of meat from goat kids (*n* = 30 samples for each experimental group). Results are expressed as ng/g of meat.

		Ageing Time						
	Group	Day 1	Day 3	Day 7	SEM ^1^	*p*-Value		
						A ^2^	T ^3^	A × T
Benzaldehyde	CON	19.99 ^Aa^	25.92 ^ABb^	30.64 ^B^	1.84	0.418	<0.0001	0.520
	RLE	20.90 ^a^	24.67 ^ab^	27.30 ^b^				
Propanal	CON	30.03 ^A^	38.24 ^B^	45.94 ^C,X^	1.05	0.007	<0.0001	<0.0001
	RLE	31.10 ^A^	36.97 ^B^	39.03 ^B,Y^				
Pentanal	CON	323.89 ^A^	405.80 ^AB^	496.96 ^B^	42.93	0.293	0.027	0.398
	RLE	330.20	391.87	392.89				
Hexanal	CON	12,463.71 ^A^	15,886.02 ^AB^	19,064.48 ^B^	1402.65	0.125	0.002	0.541
	RLE	12,095.61	14,365.59	15,603.35				
Heptanal	CON	382.39 ^A^	469.09 ^AB^	586.73 ^B^	52.20	0.512	0.057	0.258
	RLE	409.96	485.61	458.40				
Octanal	CON	373.61 ^a^	454.90 ^ab^	574.17 ^b,x^	55.21	0.064	0.125	0.227
	RLE	352.34	416.87	378.32 ^y^				
2-octenal	CON	13.82 ^a^	16.96 ^ab^	21.18 ^b^	2.30	0.893	0.112	0.496
	RLE	15.36	18.07	17.77				
Nonanal	CON	1176.23 ^A^	1477.52 ^AB^	1802.27 ^B,x^	126.32	0.151	0.006	0.230
	RLE	1190.49	1412.41	1403.16 ^y^				
2-nonenal	CON	9.44 ^A^	12.02 ^AB,x^	14.41 ^B,X^	1.28	<0.0001	0.154	0.134
	RLE	6.55	7.68 ^y^	6.42 ^Y^				
2,4-decadienal	CON	5.95 ^a^	7.43 ^ab^	9.07 ^b,X^	1.07	0.001	0.236	0.479
	RLE	4.08	4.08	4.64 ^Y^				
Dodecanal	CON	3.07	3.94	4.66	0.63	0.516	0.141	0.872
	RLE	3.05	3.63	3.98				
2,4-dodecadienal	CON	3.39 ^a^	4.28 ^ab^	5.19 ^b^	0.47	0.599	0.087	0.216
	RLE	4.16	4.89	4.42				

^1^ Standard error of the means; ^2^ anthocyanin; ^3^ ageing time. Different letters in the same row show statistical differences during time in the same group: ^A–C^ = *p* < 0.01; ^a,b^ = *p* < 0.05. Different letters in the same column show statistical differences between groups at the same ageing time: ^X,Y^ = *p* < 0.01; ^x,y^ = *p* < 0.05. CON: control; RLE: red orange and lemon extract.

**Table 4:** Effects of anthocyanin diet addition and ageing on ketone volatile compounds of meat from goat kids (*n* = 30 samples for each experimental group). Results are expressed as ng/g of meat.

		Ageing Time						
	Group	Day 1	Day 3	Day 7	SEM ^1^	*p*-Value		
						A ^2^	T ^3^	A × T
2,3-pentanedione	CON	49.37 ^A^	60.26 ^A^	103.05 ^B^	11.13	0.268	0.001	0.411
	RLE	66.40	80.71	96.05				
2-hexanone, 4-methyl-	CON	5.02 ^A,x^	6.15 ^Aa,x^	10.52 ^Bb,x^	1.45	<0.0001	0.003	0.787
	RLE	10.27 ^a,y^	12.44 ^ab,y^	14.80 ^b,y^				
2-heptanone	CON	12.17 ^A^	15.60 ^A^	26.68 ^B^	2.34	0.762	<0.0001	0.180
	RLE	15.46 ^a^	18.56 ^ab^	22.18 ^b^				
2-heptanone, 6-methyl-	CON	5.41 ^A^	6.87 ^ABa^	11.75 ^Bb^	1.46	0.912	0.009	0.403
	RLE	6.47	7.84	9.32				
3-hepten-2-one	CON	18.10 ^A,x^	21.07 ^A,X^	36.03 ^B,X^	1.67	<0.0001	<0.0001	<0.001
	RLE	11.97 ^a,y^	14.47 ^ab,Y^	16.93 ^b,Y^				
5-hepten-2-one,6-methyl-	CON	17.04 ^A^	19.87 ^Aba,x^	33.98 ^Bb^	3.77	0.002	0.001	0.537
	RLE	27.74 ^a^	33.68 ^ab,y^	39.41 ^b^				
2-octanone	CON	12.87 ^A^	16.36 ^A^	27.98 ^B,X^	2.33	0.002	<0.001	0.052
	RLE	10.84	13.06	15.28 ^Y^				
1-octen-3-one	CON	26.64 ^a^	34.21 ^a^	58.50 ^b^	8.32	-	0.030	-
	RLE	-	-	-				
3,5-octadien-2-one	CON	5.11 ^A^	6.47 ^A^	11.07 ^B,X^	0.57	0.061	<0.0001	0.003
	RLE	5.15 ^A^	6.65 ^AB^	7.78 ^B,Y^				
3-nonanone	CON	20.97 ^A,X^	24.43 ^A,X^	41.78 ^AB,X^	2.06	<0.0001	<0.0001	<0.001
	RLE	13.08 ^Y^	15.73 ^Y^	18.40 ^Y^				
6,7-dodecanedione	CON	939.04 ^A^	1091.78 ^A^	1866.96 ^B^	186.20	0.041	<0.001	0.443
	RLE	1329.95 ^a^	1603.34 ^ab^	1917.43 ^b^				

^1^ Standard error of the means; ^2^ anthocyanin; ^3^ ageing time. Different letters in the same row show statistical differences during time in the same group: ^A,B^ = *p* < 0.01; ^a,b^ = *p* < 0.05. Different letters in the same column show statistical differences between groups at the same ageing time: ^X,Y^ = *p* < 0.01; ^x,y^ = *p* < 0.05. CON: control; RLE: red orange and lemon extract.

**Table 5:** Effects of anthocyanin diet addition and ageing on alcohol volatile compounds of meat from goat kids (*n* = 30 samples for each experimental group). Results are expressed as ng/g of meat.

		Ageing Time						
	Group	Day 1	Day 3	Day 7	SEM ^1^	*p*-Value		
						A ^2^	T ^3^	A × T
1-pentanol	CON	471.72 ^A^	549.73 ^ABa^	699.34 ^Bb^	52.25	0.321	0.001	0.833
	RLE	445.66 ^a^	526.90 ^ab^	620.18 ^b^				
1-penten-3-ol	CON	66.17 ^a^	77.15 ^ab^	98.36 ^b^	10.52	0.383	0.019	0.959
	RLE	74.13	87.50	102.69				
2-penten-1-ol	CON	9.17 ^a^	10.69 ^ab^	13.62 ^b^	1.37	0.106	0.021	0.858
	RLE	11.18	13.19	14.63				
1-hexanol	CON	127.95 ^a^	149.03 ^ab^	189.51 ^b^	20.46	0.861	0.028	0.941
	RLE	133.80	157.14	184.34				
1-hexanol, 2-ethyl-	CON	19.00 ^A^	22.12 ^AB^	28.20 ^B^	2.36	0.674	0.004	0.875
	RLE	18.75 ^a^	22.14 ^ab^	25.98 ^b^				
2-hexen-1-ol	CON	30.99 ^Aa^	36.10 ^Ab^	45.88 ^B^	1.49	-	<0.0001	-
	RLE	-	-	-				
1-heptanol	CON	24.54 ^A^	28.58 ^A^	36.35 ^B,x^	1.46	0.052	<0.0001	0.494
	RLE	23.14 ^A^	27.28 ^A^	31.98 ^B,y^				
1-octanol	CON	48.07 ^A, x^	56.02 ^ABa^	71.07 ^Bb^	5.11	0.060	0.001	0.763
	RLE	42.39 ^a,y^	50.01 ^ab^	58.70 ^b^				
1-octen-3-ol	CON	458.26 ^a^	531.59 ^ab^	676.62^b^	74.78	0.061	0.017	0.976
	RLE	564.40 ^a^	667.16 ^ab^	785.45 ^b^				
2-octen-1-ol	CON	22.68	26.37	33.23	3.87	0.627	0.137	0.710
	RLE	22.80	26.87	27.98				
1-nonanol	CON	1.95 ^x^	2.26 ^X^	2.94 ^X^	1.68	<0.0001	0.500	0.838
	RLE	7.73 ^y^	9.16 ^Y^	10.73 ^Y^				

^1^ Standard error of the means; ^2^ anthocyanin; ^3^ ageing time. Different letters in the same row show statistical differences during time in the same group: ^A,B^ = *p* < 0.01; ^a,b^ = *p* < 0.05. Different letters in the same column show statistical differences between groups at the same ageing time: ^X,Y^ = *p* < 0.01; ^x,y^ = *p* < 0.05. CON: control; RLE: red orange and lemon extract.

**Table 6:** Effects of anthocyanin diet addition and ageing on hydrocarbon and carboxylic acid volatile compounds of meat from goat kids (*n* = 30 samples for each experimental group). Results are expressed as ng/g of meat.

		Ageing Time						
	Group	Day 1	Day 3	Day 7	SEM ^1^	*p*-Value		
						A ^2^	T ^3^	A × T
Hydrocarbons								
heptane	CON	50.31 ^A^	63.96 ^A^	109.37 ^B^	5.20	-	<0.0001	-
	RLE	-	-	-				
octane	CON	66.52 ^A^	81.79 ^A^	139.86 ^B^	15.32	0.348	<0.001	0.725
	RLE	62.94 ^a^	75.84 ^ab^	113.87 ^b^				
2,2,4,4-tetramethyloctane	CON	5.39 ^A,X^	6.78 ^A,X^	11.59 ^B^	0.94	<0.0001	<0.0001	0.112
	RLE	10.79 ^a,Y^	12.86 ^ab,Y^	13.88 ^b^				
2,2,7,7-tetramethyloctane	CON	21.96 ^A^	27.52 ^ABa^	47.06 ^Bb^	5.68	0.047	0.011	0.268
	RLE	36.02	43.03	45.75				
pentane, 1-cyclopropyl-	CON	57.76 ^A^	75.03 ^A^	128.31 ^B,X^	9.21	0.003	<0.0001	0.081
	RLE	50.22 ^a^	60.65 ^ab^	81.22 ^b,Y^				
benzene, 1,3-dimethyl-	CON	10.79 ^A^	12.57 ^AB^	13.93 ^B^	0.73	-	0.019	-
	RLE	-	-	-				
Carboxylic acids								
ethanoic acid	CON	32.81	38.08	65.12	13.86	0.710	0.185	0.795
	RLE	40.98	49.11	58.61				
butanoic acid	CON	6.08	7.07	12.10	4.71	0.012	0.402	0.975
	RLE	15.19	18.19	21.73				
pentanoic acid	CON	5.00 ^A^	5.83 ^A^	9.97 ^B,X^	0.74	<0.0001	<0.001	0.013
	RLE	3.23	3.89	4.19 ^Y^				
hexanoic acid	CON	52.92 ^a^	61.82 ^a^	105.71 ^b^	15.47	0.384	0.055	0.480
	RLE	52.02	62.31	72.86				
heptanoic acid	CON	81.65	94.17	161.03 ^x^	34.17	0.021	0.347	0.589
	RLE	38.12	45.68	54.01 ^y^				
octanoic acid	CON	10.92 ^A^	12.73 ^A^	21.77 ^B^	1.86	0.098	<0.0001	0.262
	RLE	14.79 ^a^	17.64 ^ab^	20.80 ^b^				
nonanoic acid	CON	25.65 ^A^	29.91 ^A,x^	51.14 ^B^	2.71	0.146	<0.0001	0.023
	RLE	32.44 ^A^	38.51 ^AB,y^	45.55 ^B^				

^1^ Standard error of the means; ^2^ anthocyanin; ^3^ ageing time. Different letters in the same row show statistical differences during time in the same group: ^A,B^ = *p* < 0.01; ^a,b^ = *p* < 0.05. Different letters in the same column show statistical differences between groups at the same ageing time: ^X,Y^ = *p* < 0.01; ^x,y^ = *p* < 0.05. CON: control; RLE: red orange and lemon extract.

**Table 7:** Effects of anthocyanin diet addition and ageing on furans, lactones, sulphur compounds and thiols of meat from goat kids (*n* = 30 samples for each experimental group). Results are expressed as ng/g of meat.

		Ageing Time						
	Group	Day 1	Day 3	Day 7	SEM ^1^	*p*-Value		
						A ^2^	T ^3^	A × T
Furans								
furan, 2-ethyl-	CON	11.82	13.78	15.22	1.68	0.889	0.063	0.919
	RLE	11.48	13.72	16.20				
furan, 2-pentyl-	CON	10.55	12.32	14.44	2.29	0.014	0.106	0.897
	RLE	14.25	17.03	20.28				
Lactones								
butyrolactone	CON	5.59 ^A^	7.03 ^A^	12.03 ^B^	1.13	0.419	<0.001	0.252
	RLE	7.44	8.88	10.59				
Sulphur compounds								
dimethyl sulfone	CON	14.78	18.46	31.58	9.41	0.176	0.308	0.933
	RLE	26.44	31.79	38.19				
Thiols								
1-propenethiol	CON	80.34 ^A^	93.61 ^A^	160.07 ^B,X^	6.48	<0.0001	<0.0001	<0.001
	RLE	71.36 ^A^	85.85 ^AB^	102.55 ^B,Y^				

^1^ Standard error of the means; ^2^ anthocyanin; ^3^ ageing time. Different letters in the same row show statistical differences during time in the same group: ^A,B^ = *p* < 0.01. Different letters in the same column show statistical differences between groups at the same ageing time: ^X,Y^ = *p* < 0.01. CON: control; RLE: red orange and lemon extract.

**Table 8:** Effects of anthocyanin diet addition and ageing on sensory evaluation of meat from goat kids (*n* = 30 samples for each experimental group).

		Ageing Time						
	Group	Day 1	Day 3	Day 7	SEM ^1^	*p*-Value		
						A ^2^	T ^3^	A × T
Tenderness	CON	6.84 ^a^	7.24	7.48 ^b^	0.05	0.418	0.034	0.321
	RLE	6.71 ^a^	7.31	7.58 ^b^				
Juiciness	CON	7.14 ^a^	7.36	7.59 ^b^	0.06	0.268	0.044	0.611
	RLE	7.12 ^a^	7.42	7.56 ^b^				
Sweetness	CON	6.02	6.15	6.14	0.04	0.662	0.415	0.180
	RLE	6.21	6.33	6.08				
Unpleasant taste	CON	4.01	3.95	3.84	0.03	0.812	0.889	0.603
	RLE	4.20	3.88	3.92				
Unpleasant odor	CON	4.51	4.44	4.28	0.04	0.488	0.358	0.757
	RLE	4.35	4.61	4.39				
Meaty odor	CON	7.46 ^a^	7.71	8.12 ^b,x^	0.06	0.034	0.012	0.042
	RLE	7.12	7.24	7.45 ^y^				
Overall assessment	CON	7.42 ^a^	7.51	8.02 ^b^	0.07	0.761	0.022	0.449
	RLE	7.32 ^a^	7.41	8.10 ^b^				

^1^ Standard error of the means; ^2^ anthocyanin; ^3^ ageing time. Different letters in the same row show statistical differences during time in the same group: ^a,b^ = *p* < 0.05. Different letters in the same column show statistical differences between groups at the same ageing time: ^x,y^ = *p* < 0.05. CON: control; RLE: red orange and lemon extract.

## Data Availability

All data is presented in the manuscript.
